# Estradiol and testosterone associated with risk of breast cancer: A meta-analysis

**DOI:** 10.5937/jomb0-50871

**Published:** 2024-11-16

**Authors:** Yanqing Liu, Yujuan Kang, Xiaofei Li, Nina Qu

**Affiliations:** 1 Yantai Yuhuangding Hospital, Breast Surgery Department, Shandong, China; 2 Yantai Yuhuangding Hospital, Ultrasound Department, Shandong, China

**Keywords:** estradiol, testosterone, breast cancer, meta-analysis, estradiol, testosteron, rak dojke, meta-analiza

## Abstract

**Background:**

This paper aimed to investigate the correlation between estradiol and testosterone in patients with breast cancer.

**Methods:**

The research papers on the correlation between estradiol and testosterone on the risk of breast cancer were searched and collected. The time limit is that each database was established until December 2023. After screening, the modified Jadad scale was used to evaluate the quality of the research literature. NoteExpress 3.2 was used for literature management, and Excel 2003 was used for data collection and extraction. Statistical analysis was performed using RevMan 5.4.1 software to determine whether there was heterogeneity in the study according to the size of the Q test (P-value), and then the OR value of combined effects was calculated using fixed or random effects models, and forest maps were drawn. At the same time, papers with the greatest weight were excluded for sensitivity analysis, and the literature bias was evaluated by drawing a funnel plot.

**Results:**

A total of 628 pieces of research were retrieved, and 11 case-control trials met the criteria for inclusion. Meta-analysis results showed that the level of E2 in breast cancer patients was higher than that in the non-breast cancer control group, but the difference was not statistically significant (OR=121.56, 95%CI (-3.32-264.44), P=0.06). The level of E2 in premenopausal patients with breast cancer was higher than that in the non-breast cancer control group, but the difference was not statistically significant (OR=8.26, 95%CI (-2.83-19.34), P=0.14). The level of E2 in postmenopausal patients with breast cancer was higher than that in the non-breast cancer control group, and the difference was statistically significant (OR=20.36, 95%CI (7.04-33.68), P=0.003). Preoperative T level was higher in patients with breast cancer than in the non-breast cancer control group, but the difference was not statistically significant (OR=14.77, 95%CI (-14.11-43.65), P=0.32). The T level before and after surgery in breast cancer patients was higher than that in the non-breast cancer control group, and the difference was statistically significant (OR=12.91, 95%CI (4.43-21.39), P=0.003). Sensitivity analysis showed that the combined effect size results were stable and reliable OR (95%CI) was 24.41 (10.21~38.61), P=0.0007. Funnel plot results showed publication bias.

**Conclusions:**

There is a positive correlation between the levels of estradiol and testosterone and the occurrence and development of breast cancer after menopause.

## Abstract (sr)

**Uvod:**

Ovaj rad je imao za cilj da istraži povezanost između estradiola i testosterona kod pacijentkinja sa rakom dojke.

**Metode:**

Pretraženi su i prikupljeni istraživački radovi o korelaciji između estradiola i testosterona na rizik od raka dojke. Vremenski okvir je bio da su baze podataka ustanovljene do decembra 2023. godine. Nakon selekcije, korišćena je modifikovana Jadad skala za ocenu kvaliteta istraživačke literature. NoteExpress 3.2 je korišćen za upravljanje literaturom, a Excel 2003 za prikupljanje i ekstrakciju podataka. Statistička analiza je izvršena korišćenjem softvera RevMan 5.4.1 kako bi se utvrdilo da li postoji heterogenost u studiji prema veliččini Q testa (P-vrednost), a zatim je OR vrednost kombinovanih efekata izračunata korišćenjem modela fiksnih ili nasumičnih efekata, i nacrtane su forest mape. Istovremeno, radovi sa najvećom težinom su isključeni radi analize osetljivosti, a pristrasnost literature je procenjena crtanjem grafikona toka.

**Rezultati:**

Ukupno je pretraženo 628 istraživačkih radova, a 11 studija slučaj-kontrola je ispunilo kriterijume za uključivanje. Rezultati meta-analize su pokazali da je nivo E2 kod pacijentkinja sa rakom dojke bio viši nego u kontrolnoj grupi bez raka dojke, ali razlika nije bila statistički značajna (OR=121,56, 95%CI (-3,32-264,44), P=0,06). Nivo E2 kod premenopauzalnih pacijentkinja sa rakom dojke bio je viši nego u kontrolnoj grupi bez raka dojke, ali razlika nije bila statistički značajna (OR=8,26, 95%CI (-2,83-19,34), P=0,14). Nivo E2 kod postmenopauzalnih pacijentkinja sa rakom dojke bio je viši nego u kontrolnoj grupi bez raka dojke, i razlika je bila statistički značajna (OR=20,36, 95%CI (7,04-33,68), P=0,003). Preoperativni T nivo je bio viši kod pacijentkinja sa rakom dojke nego u kontrolnoj grupi bez raka dojke, ali razlika nije bila statistički značajna (OR=14,77, 95%CI (-14,11-43,65), P=0,32). Nivo T pre i posle operacije kod pacijentkinja sa rakom dojke bio je viši nego u kontrolnoj grupi bez raka dojke, i razlika je bila statistički značajna (OR=12,91, 95%CI (4,43-21,39), P=0,003). Analiza osetljivosti je pokazala da su rezultati kombinovane veličine efekta stabilni i pouzdani OR (95%CI) je bio 24,41 (10,21~38,61), P=0,0007. Rezultati grafikona toka su pokazali pristrasnost u publikacijama.

**Zaključak:**

Postoji pozitivna korelacija između nivoa estradiola i testosterona i pojave i razvoja raka dojke nakon menopauze.

## Introduction

Based on statistics, approximately 1.67 million new cases of breast cancer are reported worldwide annually, with approximately 525,000 deaths attributed to the disease [1] [2]. The exact cause of breast cancer remains unclear, but it is believed to be influenced by various factors such as culture, genetics, diet, and environment [3] [4] [5] [6]. Research indicates that breast cancer is a hormone-responsive tumour, and numerous studies [7] [8] [9] [10] have suggested a correlation between endogenous and exogenous hormones and the development of breast cancer. However, there is ongoing debate regarding the association between circulating estrogen levels and breast cancer risk in both postmenopausal and premenopausal women [11] [12] [13].

With the continuous increase in mortality and incidence rates of breast cancer, particularly among younger individuals, there is a pressing need to investigate hormone-related risk factors. Understanding these factors not only aids in comprehending the etiology of breast cancer but also facilitates the implementation of appropriate preventive and intervention measures. Moreover, it is crucial to consider various factors such as race, region, lifestyle, and dietary habits, as they can influence female sex hormone levels, which exhibit significant heterogeneity. However, the limited sample sizes in existing studies necessitate further confirmation through large-scale clinical trials. Consequently, this study aims to employ a meta-analysis approach to quantitatively assess the association between female sex hormone levels and breast cancer incidence, focusing on the comparison between pre- and postmenopausal stages.

## Materials and methods

### Material sources and retrieval strategies

Computer-based searches were conducted on various databases such as China Knowledge Net, Wanfang, VIP Chinese sci-tech journals, Chinese biomedical, Pubmed, Web of Science, Cohrane Library, and others. The search duration for each database was from their respective establishment dates to December 2023. The search terms used in Chinese were »estradiol«, »testosterone«, »breast cancer«, »estrogen«, etc. To expand the search, synonyms such as »Estradiol«, »testosterone«, »breast cancer«, and »estrogen« were used as English keywords, and these keywords were linked with »AND«.

### Criteria for inclusion and exclusion of documents

Criteria for literature inclusion encompass the following aspects: (1) the literature examined should pertain to a randomized controlled trial; (2) the experimental group should consist of women diagnosed with breast cancer by means of pathology, while the control group should consist of women without breast cancer; (3) both groups should exclude individuals with any other significant illnesses impacting their overall physical condition; (4) within the past year, participants should not have undergone any oral hormone treatment; (5) for the premenopausal group, it is necessary for their menstrual cycles to be reasonably regular, whereas the postmenopausal group should meet the menopausal definition as outlined in the guidelines.

Exclusion criteria for the literature review encompassed the following aspects: (1) elimination of redundant and unrelated studies and reviews; (2) exclusion of non-randomized controlled trials; (3) removal of studies conducted solely on animal subjects; (4) omission of studies with inconsistent outcome indicators; (5) exclusion of studies with missing or incomplete data. Additionally, studies that were unusable or showed significant errors were also disregarded.

### Literature screening and data extraction

The literature screening process involved two research group members who independently applied the methods of literature inclusion and exclusion. Initially, they reviewed the title and abstract of each article, and if necessary, they accessed the full text. In case of any disagreements, consultation with third-party experts was sought. Data extraction was based on a pre-established literature feature table, focusing on the design type, total sample size, test group sample size, control group sample size, outcome index, and other relevant details from the selected documents.

### Document quality evaluation

The quality assessment of the literature included in this study was conducted using the modified Jadad scale. This scale consisted of seven criteria, which evaluated various aspects such as randomization, blinding, and handling of withdrawal and loss of follow- up. Studies that scored 0 were not included in the analysis, while those scoring 1–3 were considered low-quality studies, and those scoring 4–7 were deemed high-quality studies.

### Statistical analysis

Literature management was conducted using NoteExpress3.2 software, while Excel2003 software was utilized to collect and extract literature data. Meta-analysis was performed using Revman5.4.1 software, with the Q test (P value) employed to assess the heterogeneity of the extracted data and the I2 value used to evaluate the degree of heterogeneity. If the P value exceeded 0.10 or the I2 value was less than or equal to 50%, it indicated the absence of heterogeneity, and the fixed effect model (FEM) analysis was conducted. Conversely, if the P value was less than or equal to 0.10 or the I2 value exceeded 50%, the random effect model (REM) analysis was employed. To analyze the data and interpret the results, the odds ratio (OR) and its 95% confidence interval (CI) were utilized, and a forest plot was generated. Sensitivity analysis was applied to assess the stability of the meta-analysis outcomes, and a funnel plot was employed to evaluate publication bias. The significance level was set at =0.05 (two-tailed).

## Results

### Results of literature retrieval

**Figure 1:**
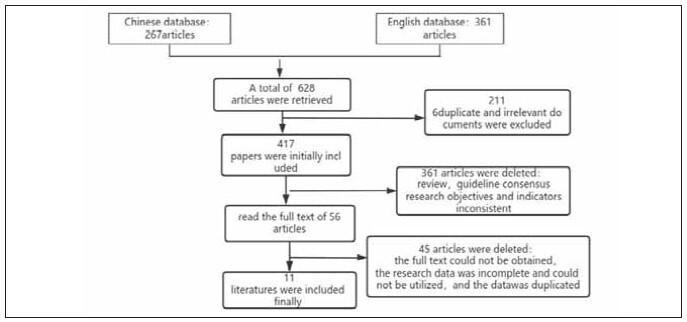
Flow chart of literature screening.

Based on the article retrieval strategy, an initial search was conducted in various databases, including China Knowledge Network, Wanfang Database, VIP Chinese Sci-tech Journals Database, China Biomedical Database, Pubmed, and Cochrane Library. 628 relevant articles were identified, and duplicates within each database were excluded. Subsequently, 11 articles were selected for inclusion after evaluating the title, abstract, and full text [14] [15] [16] [17] [18] [19] [20] [21] [22] [23] [24]. Please refer to Figure 1 for an overview of the literature screening process.

### Basic characteristics and quality evaluation of literature

The baseline information primarily consisted of variables such as gender, age, duration of illness, treatment regimen, outcome measures, and others. The revised Jadad scale was employed to assess the quality of the 11 studies included, which is illustrated in Table 1 [14] [15] [16] [17] [18] [19] [20] [21] [22] [23] [24]. All studies included in this meta-analysis were involved in a retrospective study design.

**Table 1:** Basic characteristics and quality evaluation table of documents.

First author	Year of<br>publication	Research<br>type	Sample size<br>(example)		Age		Outcome<br>index	Jadad<br>scores
			Test<br>group	Control<br>group	Test<br>group	Control<br>group		
Li Dandan [14]	2012	Case-control	107	111	59	58	Postmenopausal E2	4
Li Dandan [15]	2015	Case-control	274	279	60.7	61.1	Postmenopausal E2/<br>postmenopausal T	4
Chen Xianrong [16]	2019	Case-control	110	58	50.3	49.5	E2	4
Shi Ying [17]	2012	Case-control	35	30	44.29	42.57	E2	4
Miao Suyu [18]	2015	Case-control	54	37	39.94	39.03	Premenopausal E2	4
Huang Ruofei [19]	2018	Case-control	63	57	57.20	56.63	Postmenopausal E2/<br>postmenopausal T	4
Ma Ruilan [20]	2013	Case-control	75	78	43.4	43.2	Premenopausal E2/<br>premenopausal T	4
Xu Hong [21]	2004	Case-control	41	100	/	/	E2	4
Ma Ruilan [22]	2007	Case-control	105	100	45.3	46.5	Premenopausal and<br>postmenopausal E2/<br>premenopausal and<br>postmenopausal T	5
Lin Danli [23]	2020	Case-control	31	32	58.16	57.92	Postmenopausal E2/<br>postmenopausal T	4
Kang Xinmei [24]	2014	Case-control	90	32	/	/	Postmenopausal E2/<br>postmenopausal T	4

### Meta-analysis results mate analysis of the correlation between E2 and breast cancer

**Figure 2:**
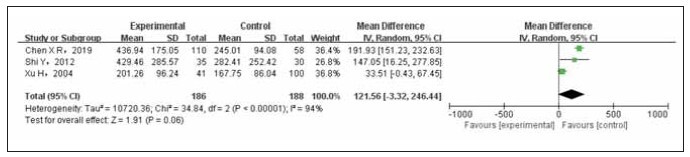
A forest map of 2E2 levels compared between the experimental and control groups.

Three studies have been conducted to examine the relationship between E2 levels and breast cancer. The test group consisted of 186 cases, while the control group had 188 cases. After conducting a heterogeneity test on the included studies, it was found that there was statistical heterogeneity among them. A Random Effects Model (REM) was used to combine the data from these studies to address this heterogeneity. The meta-analysis results indicated that breast cancer patients had higher levels of E2 compared to non-breast cancer controls, although the difference was not statistically significant (OR=121.56, 95% CI (-3.32 to 264.44), p=0.06), as illustrated in Figure 2.

### Meta analysis of the relationship between premenopausal E2 and breast cancer

**Figure 3:**
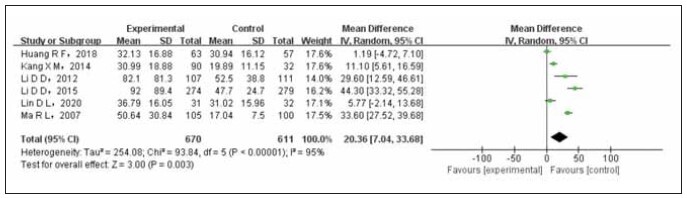
Forest map of premenopausal E2 levels compared between the experimental group and the control group.

A total of 3 articles compared the correlation between premenopausal E2 levels and breast cancer. Among them were 234 cases in the test group and 215 cases in the control group. The heterogeneity of the included literature was tested, which showed that there was statistical heterogeneity among different literature studies, so REM was used to combine the literature data. The meta-analysis showed that premenopausal E2 levels in breast cancer patients were higher than those in non-breast cancer controls, but the difference was not statistically significant (OR=8.26,95%CI (-2.83–19.34), PP0.14), as shown in Figure 3.

### Meta analysis of the relationship between postmenopausal E2 and breast cancer

**Figure 4:**
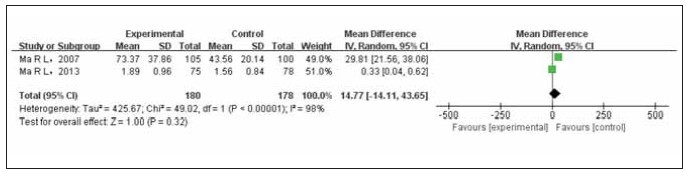
Forest map of postmenopausal E2 levels compared between the experimental and control groups.

A total of 6 studies conducted a comparison between postmenopausal E2 levels and breast cancer. The test group comprised 670 cases, while the control group had 611 cases. A heterogeneity test was conducted on the included studies, revealing statistical heterogeneity among them. Therefore, a randomeffects model (REM) combined the data. The results of the meta-analysis demonstrated a significant elevation in E2 levels among postmenopausal breast cancer patients compared to non-breast cancer controls (OR=20.36, 95%CI (7.04–33.68), PP0.003). Figure 4 illustrates this finding. The studies included in the analysis were as follows: Marilan [22] (case-control, 10510045.346.5, premenopausal E2/premenopausal T5), Lin Danli [23] (case-control, 2020, premenopausal E2/premenopausal T4), and Kangxinmei [24] (case-control, 2014, postmenopausal E2/postmenopausal T4, 9032 cases).

### Meta analysis of the relationship between premenopausal T and breast cancer

**Figure 5:**
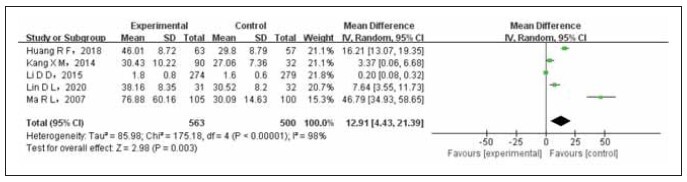
Forest map of premenopausal T level compared between the experimental group and the control group.

Two studies were conducted to compare the association between premenopausal T levels and breast cancer. The test group comprised 180 cases, while the control group included 178 cases. A heterogeneity test was performed to assess the heterogeneity among the studies, revealing statistical heterogeneity. To address this, the Random Effects Model (REM) combined the data from the different studies. The results of the meta-analysis indicated that breast cancer patients had higher preadipose T levels compared to non-breast cancer controls, although the difference was not statistically significant (OR=14.77, 95%CI (-14.11–43.65), Prun0.32), as illustrated in Figure 5.

### Meta analysis of the relationship between postmenopausal T and breast cancer

**Figure 6:**
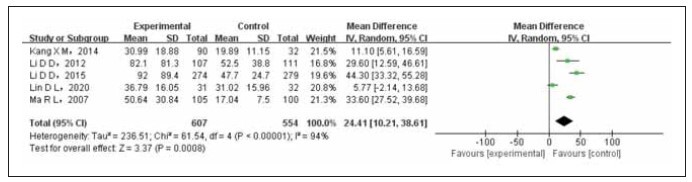
Forest map of postmenopausal T level compared between the experimental group and the control group.

A total of 5 literature sources have compared the association between levels of postmenopausal T and breast cancer. The test group consisted of 563 cases, while the control group had 500 cases. The included literature was subjected to a heterogeneity test, revealing statistical heterogeneity across different studies. Consequently, the random-effects model (REM) was employed to merge the data from these literature sources. Meta-analysis results indicated that breast cancer patients had higher levels of pre- and postmenopausal T compared to non-breast cancer controls, and this difference was statistically significant (OR=12.91, 95% CI (4.43–21.39), Prun0.003), as depicted in Figure 6.

### Sensitivity analysis

**Figure 7:**
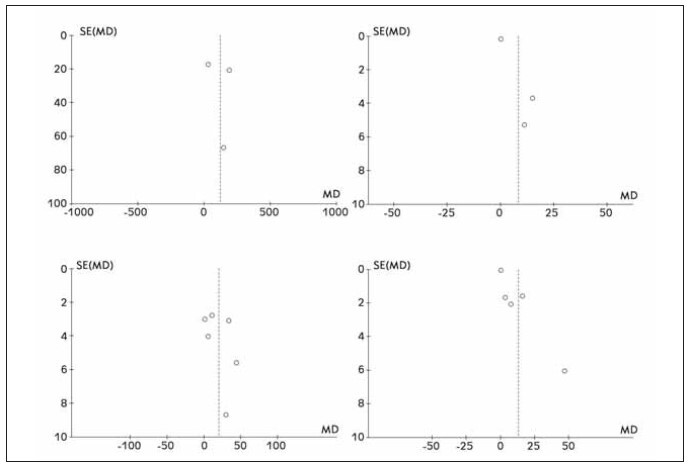
Forest map of sensitivity analysis.

The sensitivity analysis was conducted using the outcome index of postmenopausal E2 level, which had the highest number of referenced literature sources. To ensure the reliability of the results, the literature with the largest proportion (both articles had the same proportion) was removed. This resulted in an OR (95%CI) of 24.41 (10.21, 38.61) and a literature effect of 0.0007, confirming the credibility of the study findings, as illustrated in Figure 7.

### Literature bias examination

**Figure 8:**
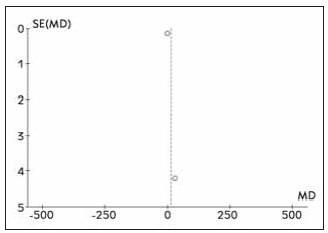
Forest funnel diagram included in the literature.

All the outcome indicators involved in this paper were biased, and the results showed that there was asymmetry in the funnel chart, indicating that there was bias. See Figure 8.

## Discussion

In recent years, as medical technology continues to advance and individuals become more conscious of their health, the incidence of breast cancer in China has been on the rise [25]. Breast cancer primarily affects the glandular epithelium of the breast, representing a common malignant tumour in clinical settings. Patients with breast cancer often exhibit painless breast masses characterized by an uneven surface, irregular edges, and a firm texture. Additionally, they may experience nipple discharge of blood or serous fluid, accompanied by itching and ulceration of the nipple skin. Ipsilateral axillary lymph nodes may also enlarge as a result. The cancer cells easily detach from the primary tumour site, leading to metastasis through the bloodstream, lymphatic system, and other routes. This poses a significant threat to the life and well-being of patients [26] [27].

The meta-analysis findings indicated that E2 levels in breast cancer patients were higher compared to non-breast cancer controls. However, statistically significant differences were not observed (OR=121.56, 95%CI (-3.32 to 264.44), Prun0.06). Similarly, the level of E2 in premenopausal breast cancer patients was also higher than in non-breast cancer controls. However, these differences were not statistically significant (OR=8.26, 95%CI (-2.83 to 19.34), Prun0.14). Conversely, postmenopausal breast cancer patients exhibited significantly higher levels of E2 when compared to non-breast cancer controls, and this difference was statistically significant (OR=20.36, 95%CI (7.04 to 33.68), P<0.003). Although the premenopausal T level in breast cancer patients was higher than in non-breast cancer controls, these differences were not statistically significant (OR=14.77, 95%CI (-14.11 to 43.65), Prun0.32). However, both pre and postmenopausal breast cancer patients showcased significantly higher T levels than non-breast cancer controls, and these differences were statistically significant (OR=12.91, 95%CI (4.43 to 21.39), P<0.003). Sensitivity analysis helped confirm the stability and reliability of the combined effect, with an OR _ (95%CI) of 24.41 (10.21 to 38.61) and a P-value of 0.0007. The findings of the funnel chart analysis revealed the presence of publication bias.

This study has certain constraints: the meta-analysis revealed a presence of bias, possibly due to the extensive duration and inadequate sample size of the included literature. Furthermore, the retrieval process solely relied on Chinese and English databases, contributing to potential sampling bias resulting from the selective collection of literature within these databases. This selective collection in each database further contributes to the bias observed in the study’s findings.

## Conclusion

In conclusion, this meta-analysis demonstrates a positive correlation between elevated levels of estradiol and testosterone in postmenopausal women and the incidence and progression of breast cancer. Nevertheless, future research should focus on multicenter studies with large sample sizes and homogenous case-control groups.

## Dodatak

### Registration and protocol

The review was not registered.

### Funding

The present study was supported by the Shandong Provincial Natural Science Foundation (No. ZR2021MH398).

### Author contributions

Yanqing Liu and Yujuan Kang: study design, data analysis, drafting the manuscript, and revision of the manuscript; Xiaofei Li and Nina Qu: data collection and analysis, drafting the manuscript, investigation. All authors read and approved the final version of the manuscript.

These authors contribute equally to the present work.

### Acknowledgements

None.

### Availability of data

All data generated or analyzed in this study are included in the present manuscript.

### Conflict of interest statement

All the authors declare that they have no conflict of interest in this work.
